# Identification of Two Subtypes and Prognostic Characteristics of Lung Adenocarcinoma Based on Pentose Phosphate Metabolic Pathway-Related Long Non-coding RNAs

**DOI:** 10.3389/fpubh.2022.902445

**Published:** 2022-06-21

**Authors:** Chuan Liu, Yongjie Wang

**Affiliations:** Department of Thoracic Surgery, Affiliated Hospital of Qingdao University, Qingdao, China

**Keywords:** lung adenocarcinoma, long non-coding RNAs, pentose phosphate metabolic pathway, transcription factors, immunotherapy

## Abstract

This study analyzed the differences in subtypes and characteristics of advanced lung adenocarcinoma (LUAD) patients based on the pentose phosphate metabolic pathway-related long non-coding RNAs (lncRNAs), along with their potential regulatory mechanisms. Using the expression profiling and corresponding clinical information of LUAD patients from Gene Expression Omnibus (GEO) and the Cancer Genome Atlas (TCGA). Differential pathway scores between normal and tumor samples from TCGA were identified by rank-sum tests. Pearson correlation coefficients between pentose phosphate scores of the pentose phosphate samples and lncRNAs of the corresponding datasets were calculated. Next, the clusterProfiler software package was used for functional annotation. Clustering of pentose phosphate-related lncRNAs from LUAD samples categorized two molecular subtypes (C1, and C2). C1 was associated with a lower pentose phosphate score and a good prognosis; the C2 showed a higher pentose phosphate score and was related to poorer prognoses. The C2 was markedly associated with energy metabolic pathways. The expression of most immune cells were markedly higher in C1 subtype. Some crucial immune checkpoints, including CTLA4, CD274, and CD47, were also significantly upregulated in C1 subtype, leading to a higher score of clinical effect on the C1 subtype. Finally, one TF, BACH1, was found to be significantly upregulated in C1 subtypes; the pathways activated by this TF may be associated with tumor progression and poor prognoses. LUAD typing based on pentose phosphate metabolic pathway-related lncRNAs was confirmed. Differences in characteristics between C1 and C2 subtypes improved the current LUAD detection and treatment.

## Introduction

Among the malignancies associated with the highest mortality, worldwide, lung cancer leads (1). The most frequent lung cancer histological subtype, accounting for ~50% of all the cases, is lung adenocarcinoma (LUAD). Patients with LUAD are at a high risk of distant metastases at each stage of the disease progression (2), along with high malignancy and poor prognoses (3, 4). Treatment of LUAD is based on grading and stage, mainly determined using the patients' characteristics and the pathological assessment of the tumor histology (5).

Especially, for metastatic LUAD patients, traditional therapy such as surgery and radiotherapy do a few efficacy for them. Targeted therapy or immunotherapy could be a superior choice for metastatic patients. Targeted therapeutic drugs such as anaplastic lymphoma kinase (ALK) inhibitors (6) and epidermal growth factor receptor (EGFR) inhibitors (7) have showed a promising performance in clinical trials for treating lung cancer patients with ALK or EGFR mutations. In the case of metastatic patients without these specific mutations, immunotherapy may be a wise choice. For example, programmed cell death protein 1/programmed cell death ligand 1 (PD-1/PD-L1) blockade therapy is considered as the most efficient in many cancer types (8). By the in combination with other treatments such as radiotherapy, chemotherapy or targeted therapy, the overall survival could be prolonged (9). However, not all patients can benefit from these therapies because of the individual differences and responses. Therefore, it is necessary to explore key molecular biomarkers for understanding further tumorigensis mechanism and assisting personalized treatment in lung cancer.

Long non-coding RNAs (lncRNAs) are transcripts with a length of more than 200 nucleotides; they do not encode for proteins (10). Their functions are related to the subcellular localization of lncRNAs. Typically, lncRNAs in the nucleus can repress or activate the expression of their target genes by binding to them directly. In addition, they regulate gene expression through histone modification or recruitment of TFs. LncRNAs in the cytoplasm function as competitive endogenous RNAs through their interactions with corresponding miRNAs for the regulation of target gene expression (11–13). Several studies over the last decade show that lncRNAs exert different biological functions (14, 15). Aberrant expression of lncRNAs may result in a substantial effect on cancer progression, including cellular migration, proliferation, metastasis, and invasion (16).

Metabolism-related lncRNAs have been demonstrated to play an important role in regulating cancer metabolism and the crosstalk between cancer metabolism and tumor microenvironment (17). Previous studies have also discovered a series of lncRNA biomarkers or signatures related to metabolism for lung cancer based on bioinformatics analysis (18–20). However, limited research has explored molecular subtypes and prognostic biomarkers based on lncRNAs related to pentose phosphate pathway that is one of metabolism pathways involved in cancer development (21). PTTG3P, a lncRNA involved in the pentose phosphate pathway, is attenuated in non-small cell lung cancer, thereby leading to a poor prognosis (22). However, the specific regulatory mechanisms of LUAD and pentose phosphate-related lncRNAs remain unclear.

Herein, we systematically investigated the correlation of pentose phosphate-related lncRNAs with the enrichment score of pentose phosphate pathway (pentose phosphate score) in patients with advanced stage (stages IV, III, and II) and examined their associated signaling pathways, along with their clinical significance.

## Materials and Methods

### Expression Profile Data

The workflow of this study was shown in Supplementary Figure 1. The clinical information and corresponding RNA sequencing (RNA-seq) data of lung adenocarcinoma samples were obtained from The Cancer Genome Atlas (23) (TCGA, https://portal.gdc.cancer.gov/), named as TCGA dataset. In addition, expression data and clinical information of GSE30219 dataset (24) were extracted from Gene Expression Omnibus (GEO) database (https://www.ncbi.nlm.nih.gov/geo/).

### Metabolism-Related Genes

Using the R package, Immuno-Oncology Biological Research (IOBR), 114 metabolism-related pathways with gene lists were extracted (Supplementary Table 1) (25).

### Data Pre-processing

The RNA-seq data from TCGA was preprocessed in the following manner: (1) samples having incomplete survival information were excluded; samples of patients in stages II, III, or IV were retained; (2) ENSG was matched to GeneSymbol. The GSE30219 dataset was preprocessed as following: (1) the normalized dataset was downloaded; (2) samples with complete information on survival time and survival status were retained; (3) only the pentose phosphate samples of patients in stages IV, III, and II were retained; (4) GPL570 was re-annotated and the probes were then transformed to gene symbols.

### Acquisition of the LncRNA Expression Profiles

The gene transfer format (GTF) file (version: V32) was obtained from the GENCODE website (https://www.gencodegenes.org/). TCGA expression profiles and GSE30219 data were divided into mRNAs or lncRNAs according to the annotations in the file.

### Identification of Hubs in the Pentose Phosphate Pathway

Scores for metabolic pathways were calculated separately for TCGA and GSE30219 samples by single sample gene set enrichment analysis (ssGSEA) (26). Differences in scores between normal and tumor samples were identified using Wilcoxon test. Subsequently, prognosis-related metabolic pathways in TCGA and GSE30219 datasets were identified by univariate Cox analyses. Pentose phosphate pathway was identified in both two datasets and was considered as an important metabolic pathway for further analysis.

### Identification of Pentose Phosphate-Related LncRNAs

Pearson correlation coefficients between pentose phosphate scores of the pentose phosphate samples and the lncRNAs of the corresponding datasets, along with their corresponding *P*-values were calculated. Filtering for significance was performed based on the set thresholds of |correlation (cor)| > 0.25 and *P* < 0.05.

### Identification of Pentose Phosphate-Related LncRNA Subtypes

The samples were clustered and typed by constructing a consistency matrix using ConsensusClusterPlus (27). The pentose phosphate-related lncRNA subtypes of the samples were obtained based on the pentose phosphate score-related lncRNAs. In addition, the Kaplan-Meier (KM) algorithm was executed and euclidean was set as the distance metric to perform 500 bootstraps, with each bootstrap process incorporating 80% of the patients belonging to the training set. The best classification was evaluated by computing the consistency matrix and the consistency cumulative distribution function (CDF), after setting the number of clusters between two and 10.

### Gene Set Enrichment Analysis and Functional Annotation

The “GSEA” (26) package was employed to study the pathways of different molecular subtypes in biological processes. All candidate gene sets of KEGG pathways from the Molecular Signatures Database (MSigDB) database (28) were used to perform GSEA. Significantly enriched pathways were screened under the criteria of *P* < 0.05 and *Q* < 0.25. In addition, the clusterProfiler package (29) was used for functional annotation. Limma R package was used to identify differentially expressed genes (DEGs) between two subtypes (30). DEGs with |log2(fold change)| > 1 and adjust *P* < 0.05 were screened.

### Analysis of TF Activity

TF activity was scored according to the method developed by Garcia-Alonso (31) and TF activation levels were compared between clusters by analysis of variance (ANOVA). The screening criterion for significantly differentially expressed TFs was set at *P* < 0.05.

### First-Order Partial Correlation Analysis

Analysis using first-order partial correlation was performed to evaluate the correlation of glycolysis-related genes and glycolysis scores with lncRNAs. Assuming that glycolysis score is x and glycolysis-related gene expression is y, the first-order partial correlation between x and y based on lncRNAs is as follows:


$$\begin{array}{l} r_{xylncRNA}=\frac{r_{xy}-r_{xlncRNA}*r_{ylncRNA}}{\sqrt{\left(1-r_{xlncRNA}^{2}\right)*\left(1-r_{ylncRNA}^{2}\right)}} \end{array}$$

### Statistical Analysis

All statistical analysis and R packages were implemented in R software (v4.0). Log-rank test was conducted in KM survival analysis. Wilcoxon test was conducted in testing the difference between two groups. *P* < 0.05 was considered as significant.

## Results

### Molecular Typing Based on Pentose Phosphate Score-Related LncRNAs

**Figure 1:**
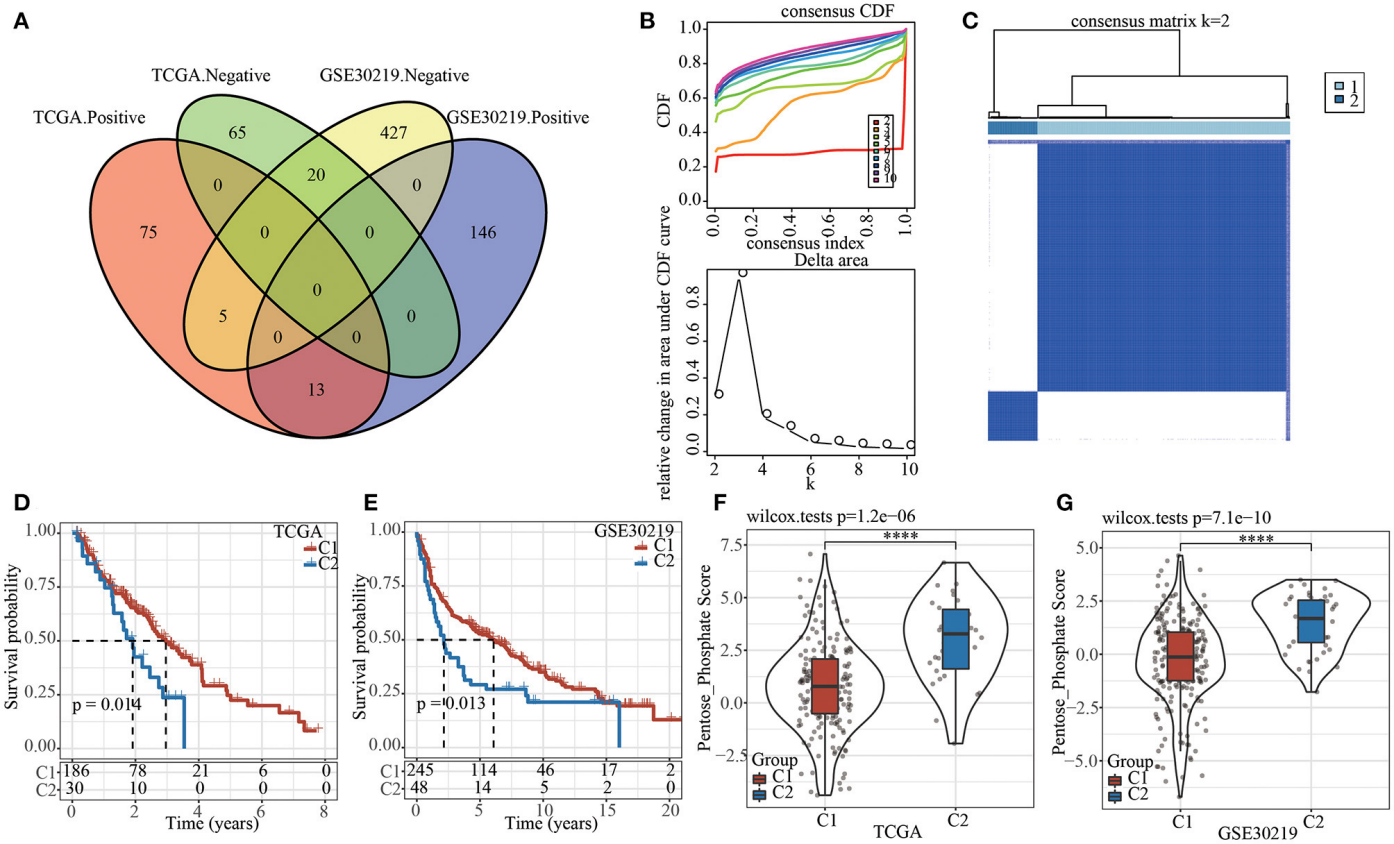
The pentose phosphate-related lncRNAs subtypes in TCGA. **(A)** Venn diagram of the intersection between the pentose phosphate activity-related lncRNAs in the two datasets; **(B)** CDF curve and CDF delta-area curve for TCGA samples. The delta-area curves for consensus clustering show the relative changes in the areas under the CDF curve for each category number “k” relative to “k-1,” where the horizontal axis represents the category number k and the vertical axis represents the relative change in area under the CDF curve; **(C)** Heat map of sample clustering in TCGA dataset at consensus k = 2. **(D)** Survival curves of two pentose phosphate-related lncRNA subtypes in TCGA dataset; **(E)** Survival curves of two pentose phosphate-related lncRNA subtypes in GSE30219 dataset; **(F)** Differences of pentose phosphate score between two molecular subtypes in TCGA dataset; **(G)** Differences of pentose phosphate score between two molecular subtypes in GSE30219 dataset. Log-rank test was conducted in **(D,E)**. Wilcoxon test was conducted in **(F,G)**. ****P < 0.0001.

Firstly, pentose phosphate pathway was identified as an important metabolic pathway related to prognosis in both TCGA and GSE30219 datasets (see Materials and methods 2.3). Correlation analysis between pentose phosphate score and lncRNA expression yielded 178 and 611 lncRNAs that were significantly associated with pentose phosphate activity in TCGA and GSE30219 datasets, respectively. A total of 38 lncRNAs were found in both two datasets (Figure 1A), but the correlations (negative and positive correlations) of five genes were found to be inconsistent in TCGA and GSE30219 datasets. Therefore, the remained 33 lncRNAs were included for identifying prognostic lncRNAs through univariate Cox analysis. Of them, 26 prognosis-related lncRNAs were screened, and their expression was used as an input for consensus clustering. Samples in TCGA dataset were clustered using ConsensusClusterPlus and the optimal number of clusters was determined based on the CDF. The CDF delta area curve shows that the clustering results were stable when the number of clusters was 2 (Figure 1B). Hence, we chose k = 2 and effectively obtained two molecular subtypes (Figure 1C). Subsequently, the prognostic characteristics for the two subtypes showed that the samples of the C1 subtype exhibited a longer survival time (Figure 1D). A similar trend was observed in GSE30219 (Figure 1E). Moreover, differential analysis of the pentose phosphate scores between the two molecular subtypes yielded lower scores in the C1 subtype relative to the C2 subtype in both TCGA and GSE30219 datasets (Figures 1F,G).

### Differential Pathways Between Two Subtypes

**Figure 2:**
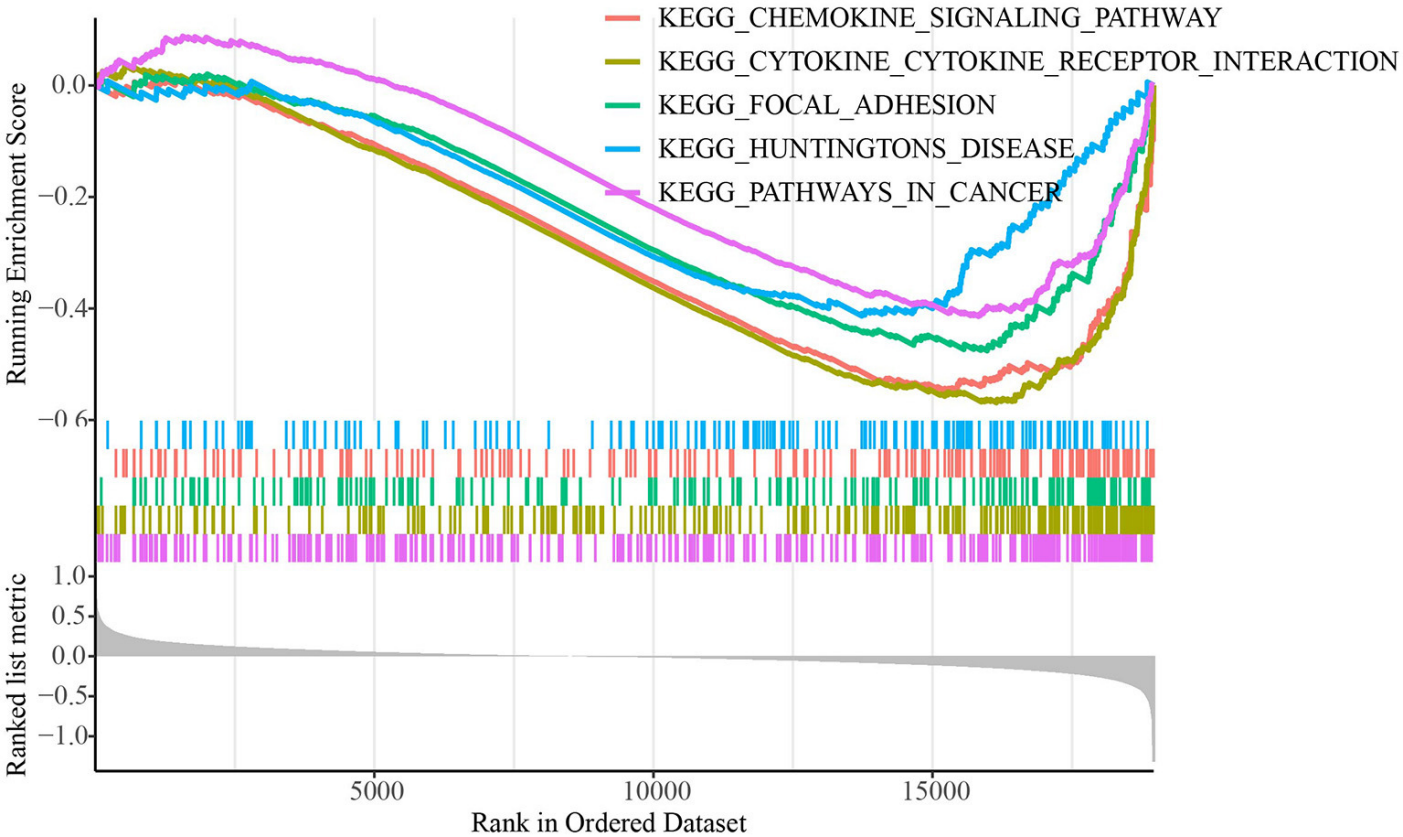
GSEA for molecular subtypes in TCGA dataset.

The TCGA dataset's distributions of key clinical features for the two molecular subtypes were examined to see if there were any changes The TCGA dataset revealed no significant variations in clinical features between the subtypes in ages, genders, and stages (Supplementary Figure 2). We used GSEA to look into the KEGG pathways connected with distinct molecular subtypes in biological processes (Figure 2). The results showed that metabolism-related pathways, including cytokine-cytokine receptor interaction, focal adhesion, and chemokine signaling pathway were significantly enriched in the C2 subtype showing a better prognosis, which indicated metabolic pathways were more activated in C2 subtype.

In addition, we performed differential expression analysis between two subtypes, and identified 266 DEGs (|log2(fold change)| > 1 and adjust *P* < 0.05), where 126 and 140 DEGs were up-regulated in C1 and C2, respectively (Supplementary Figure 3A). GO analysis revealed that these DEGs were significantly enriched in metabolic terms such as quinone metabolic process, secondary metabolic process, and prostaglandin metabolic process (Supplementary Figure 3B). KEGG analysis showed that disease-related pathways such as autoimmune thyroid disease, type I diabetes mellitus, and graft–vs.–host disease were significantly enriched (Supplementary Figure 3C).

### Mutational Characteristics of Pentose Phosphate-Related LncRNAs Subtypes

The differences in genomic alterations between these two molecular subtypes in TCGA were further examined. Correlation analysis indicated that pentose phosphate activity was not significantly associated with mutational characteristics, including the aneuploidy score, homologous recombination defects, number of segments, tumor mutation burden, and fraction altered (Supplementary Figure 4A). In addition, results of the mutation analysis suggested a significant correlation between subtypes and mutation in genes, including SPTA1, TNR, KEAP1, STK11, and FAT4, all of which had a significantly higher proportion of mutations in the C2 subtype relative to the C1 subtype (Supplementary Figure 4B).

### Pathways Related to Pentose Phosphate-Related LncRNAs Subtypes

**Figure 3:**
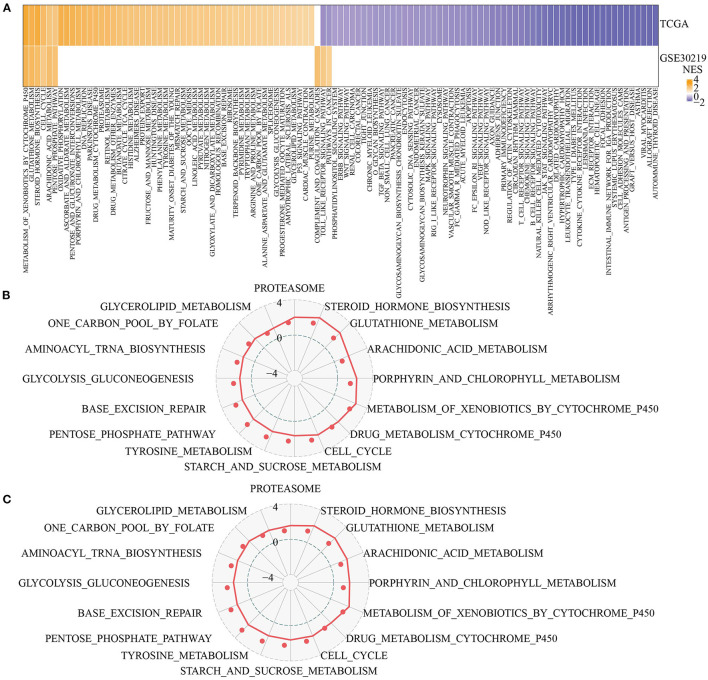
Functional enrichment analysis for pentose phosphate-related lncRNAs subtypes. **(A)** Heat map showing the normalized enrichment scores (NESs) of the hallmark pathways calculated by C1 vs. C2 (FDR <0.05); **(B,C)** Radar plot showing the NESs of hallmark pathways in TCGA **(B)** and GSE30219 **(C)** according to the GSEA by comparing C1 vs. C2.

Next, we analyzed the differentially activated pathways between the two pentose phosphate-related lncRNAs subtypes. To identify these pathways, we performed a GSEA using all candidate gene sets from the KEGG tool. We defined false discovery rate (FDR) < 0.05 as the criterion for significant enrichment of the corresponding pathway. The C2-activated pathways included VEGF signaling pathway, ECM receptor interaction, intestinal immune network for IgA production, and Toll-like receptor signaling pathway (Figure 3A). In addition, we also compared the pathways that were consistently upregulated between C1 and C2 subtypes consisting of different LUAD patients (NES > 0 in C1 vs. C2, Figures 3B,C). GSEA for different subtypes showed that patients with the C2 subtype showed significantly upregulated glutathione metabolism, porphyrin and chlorophyll metabolism, steroid hormone biosynthesis, and arachidonic acid metabolism. Thus, the lncRNAs used for molecular typing may be significantly associated with energy metabolism.

### Immune Characteristics of Pentose Phosphate-Related LncRNAs Subtypes

**Figure 4:**
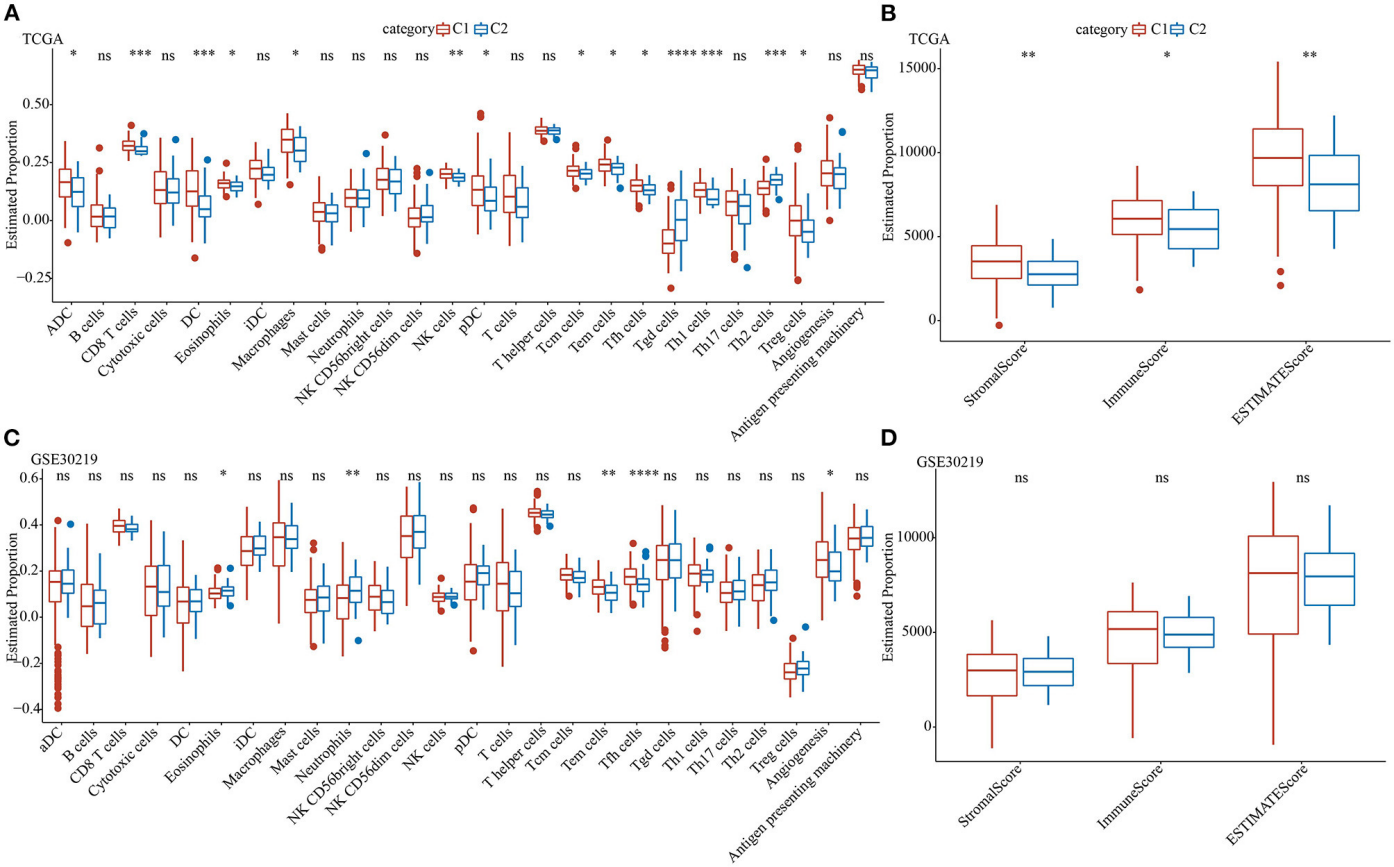
Analysis of immune characteristics of the pentose phosphate-related lncRNAs subtypes. Proportion of immune cell components for the two subtypes in TCGA **(A)** and GSE30219 **(C)**; proportions of immune cell components calculated using ESTIMATE in TCGA **(B)** and GSE30219 **(D)**. Wilcoxon test was performed. ns, no significance. *P < 0.05, **P < 0.01, ***P < 0.001, ****P < 0.0001.

To further elucidate the differences in the immune microenvironment of patients belonging to two pentose phosphate-related lncRNAs subtypes, we determined the degree of infiltration of different immune cells in patients of TCGA dataset using the expressions of gene markers of immune cells from previously published literature (32). Based on the results of the differential analysis for the expressions of markers of immune cells, the two defined molecular subtypes were found to be significantly different for most immune cells as well as some pathways. Additionally, the expressions of most immune cells in the C1 subtype were markedly greater than those in the C2 subtype, including eosinophils, effector memory T, and T helper follicular cells (Figures 4A,C). Moreover, ESTIMATE was used to assess immune cell infiltration. The findings suggested that the “ImmuneScore,” “Stromal Score,” and “ESTIMATE Score” of the C1 subtype in TCGA were substantially greater than those of the C1 subtype, thereby indicating that, overall, the C1 subtype had a high degree of immune cell infiltration (Figures 4B,D).

### Differences in the Responses to Immunotherapy Between Different Pentose Phosphate-Related LncRNAs Subtypes

**Figure 5:**
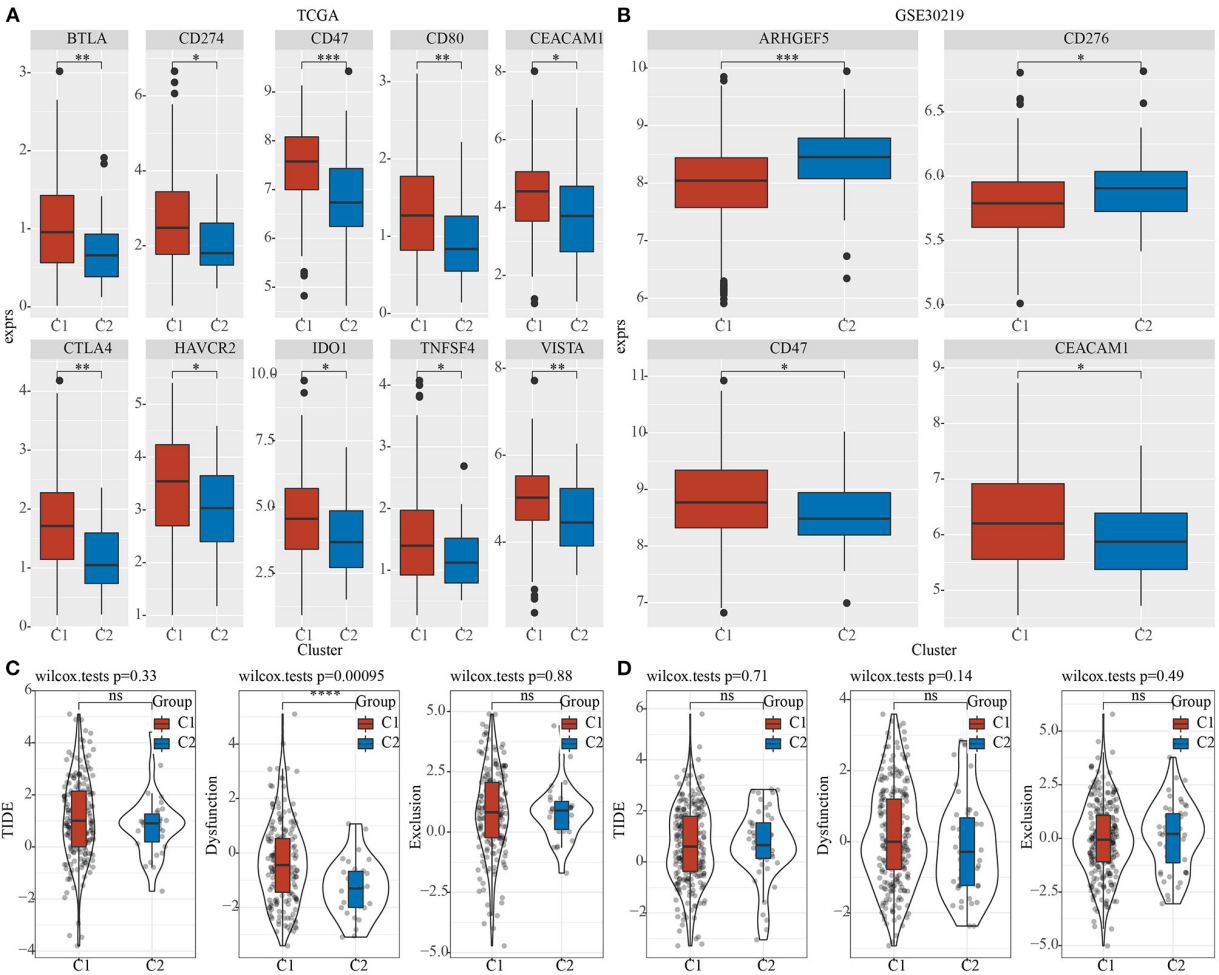
Differences in the efficacy toward immunotherapy between the two phosphate-related lncRNAs subtypes. Box plots for comparing immune checkpoints in C1 and C2 in TCGA **(A)** and GSE30219 **(B)**; **(C)** Differences in TIDE, Dysfunction, and Exclusion scores between the two molecular subtypes of TCGA dataset; **(D)** Differences in TIDE, Dysfunction, and Exclusion scores between the two molecular subtypes of the GSE30219 dataset. Wilcoxon test was performed. ns, no significance. *P < 0.05, **P < 0.01, ***P < 0.001, ****P < 0.0001.

Furthermore, differences in the responses to immunotherapy of different pentose phosphate-related lncRNAs subtypes were analyzed. First, we investigated whether there were any differences in the expressions of immune checkpoints between the two subtypes, wherein the immune checkpoints were derived from the HisgAtlas database (33). In the TCGA dataset, the expressions of the hub immune checkpoints, CTLA4, CD274, and CD47, were found to be markedly upregulated in the C1 subtype relative to those in the C2 subtype. In addition, expressions of CTLA4 and VISTA were found to be substantially high in the C1 subtype (Figure 5A); CD47 and CEACAM1 expressions were high in the C1 subtype in the GSE30219 dataset (Figure 5B). Moreover, using the TIDE (http://tide.dfci.harvard.edu/) software, the potential clinical effects of immunotherapy on the two molecular subtypes were assessed. The higher TIDE prediction score indicated a greater likelihood for immune escape, which suggested that patients were less likely to benefit from immunotherapy. We found a significantly higher dysfunction score for the C1 subtype as compared to the C2 subtype in TCGA dataset (Figure 5C). However, no significant differences were observed in the GSE30219 dataset (Figure 5D).

### Characteristics of Pentose Phosphate-Related LncRNAs

**Figure 6:**
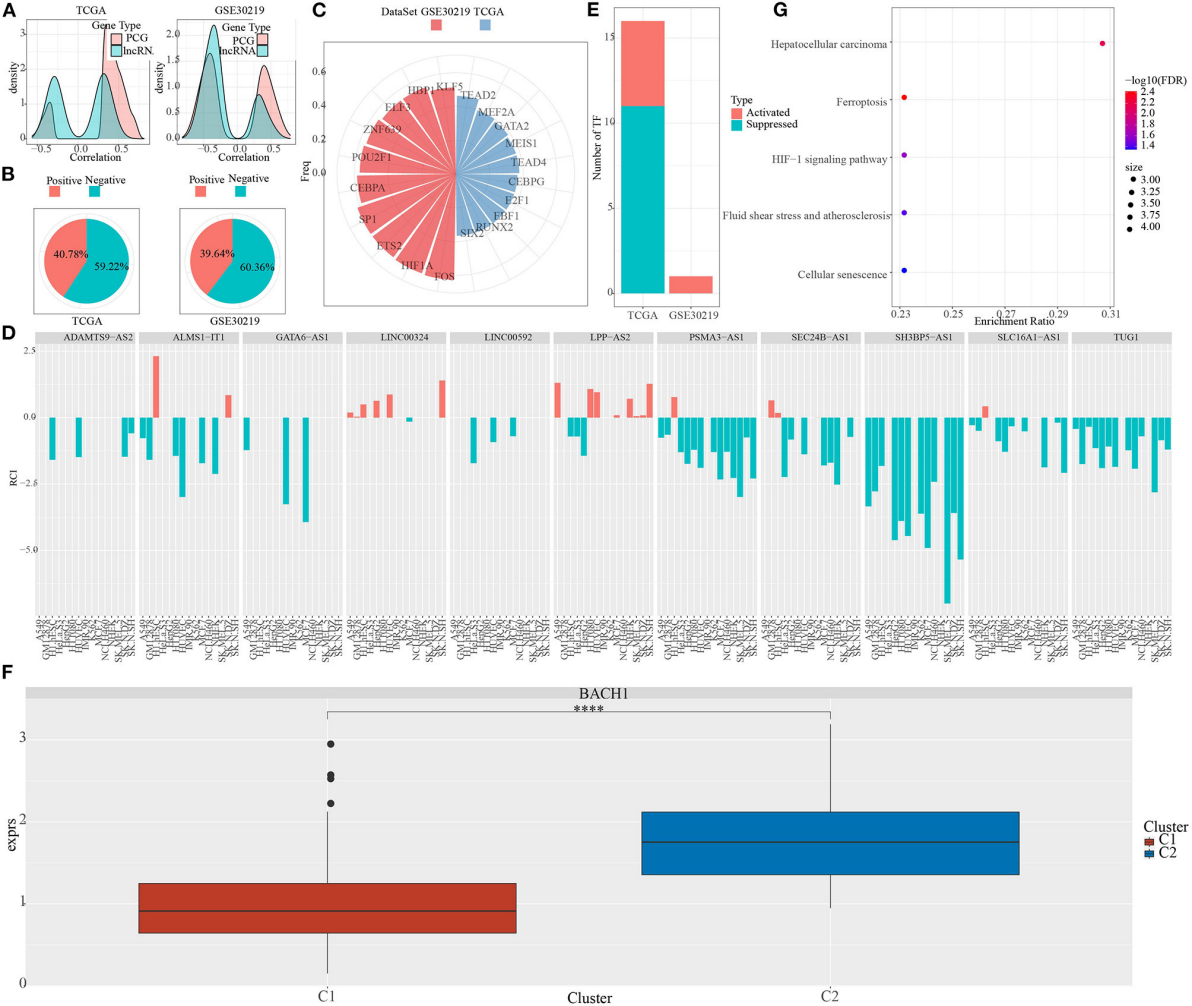
Characteristics of pentose phosphate-related lncRNAs. **(A)** Density curves for correlation coefficients of pentose phosphate-related lncRNAs with protein-coding genes. **(B)** Definition criteria for pentose phosphate-related lncRNAs in cells, where negative is defined as RCI <0 and positive as RCI > 0; **(C)** Distribution of TFs is significantly negatively associated with lncRNAs in both datasets; **(D)** Distribution of lncRNAs is consistently associated with cellular pentose phosphate activity in both datasets; **(E)** Distribution of activated and repressed TFs in the C1 subtype as compared to the C2 subtype; **(F)** Distribution of the consistently upregulated TF (BACH1) in TCGA subtypes. Wilcoxon test was performed. **(G)** Functional enrichment analysis for TFs that are consistently upregulated in the C2 subtype. ****P < 0.0001.

The majority of phosphate-related lncRNAs showed a negative association with pentose phosphate activity relative to the protein-coding genes (Figure 6A).

Next, using the LncATLAS database, we determined the cellular localization of these pentose phosphate-related lncRNAs. The majority of these lncRNAs were localized to the nucleus (Figure 6B); 59.22% of RCIs were negative (RCI <0) in TCGA and 60.36% of RCIs were negative (RCI <0) in GSE30219.

To evaluate the differences in TF activities between the two subtypes, the TF activity scores for each sample in TCGA and GSE30219 were calculated, according to the method described by Garcia-Alonso et al. (31). Further, an ANOVA test was performed to compare the TF activation levels between the two clusters in an effect to identify significantly differentially expressed TFs. A total of 91 and 51 significantly differentially expressed TFs in TCGA and GSE30219 datasets, respectively, were obtained.

Further, we investigated the association between pentose phosphate-related lncRNAs and the dysregulated TFs. Specifically, we performed a correlation analysis for the lncRNAs located in the nucleus with differentially expressed TFs; a series of TFs showing a significant negative correlation with pentose phosphate-related lncRNAs were identified (Figure 6C). In our previous study, we have reported a total of 33 pentose phosphate-related lncRNAs, of which 13 show a positive correlation with the pentose phosphate score and the remaining 20 show a negative correlation. Some of the critically significant lncRNAs were selected based on their correlation with the activity of TFs (Figure 6D). Further, the distributions of activated and repressed TFs between the C1 subtype and C2 subtype were determined. A total of 11 repressed TFs and five activated TFs were found in LUAD-TCGA, whereas one activated TF was present in the GSE30219 dataset (Figure 6E). To examine whether the activity of TFs differed between the two molecular subtypes, one TF, BACH1, that was significantly upregulated in the C2 subtype (Figure 6F) was identified. We hypothesized that these TFs, which were consistently upregulated in the C2 subtype, may be associated with the poorer prognoses of the C2 subtype. To verify our hypothesis, a functional enrichment analysis for the TFs was performed (Figure 6G), which showed significant enrichment of genes targeted by TFs in transduction cascades involved in cellular senescence, atherosclerosis, fluid shear stress, HIF-1 signaling pathway, hepatocellular carcinoma, and ferroptosis. The activation of these pathways was related to tumor progression and poor prognosis, which suggested that pentose phosphate-related lncRNAs may exert important effects in the activation and progression of some cancer-related pathways.

## Discussion

Pentose phosphate has an important function in the metabolic reprogramming of tumor cells and is usually mediated through glucose-6-phosphate dehydrogenase (G6PD) (34). However, the role of pentose phosphate in LUAD remains unclear. Thus, in this study, two molecular subtypes of LUAD, C1, and C2, were obtained based on pentose phosphate-related lncRNAs by clustering analysis of samples from TCGA and GSE datasets. The C1 subtype showed a lower pentose phosphate score and was associated with good prognoses, whereas the C2 subtype yielded a higher pentose phosphate score and was related to poor prognoses. GSEA indicated that the C2 subtype was substantially associated with pathways of energy metabolism. Unlike protein-coding genes, most pentose phosphate-related lncRNAs are negatively associated with pentose phosphate activity (13, 22). As a majority of them are localized to the nucleus, they are likely to suppress the expression of pentose phosphate by directly binding to the target genes (10, 11, 16, 35). For lncRNA-mediated regulation, we identified significantly differentially expressed TFs. These TFs may be involved in the activation of pathways related to cellular senescence, which is in turn related to tumor progression and poor prognoses in patients with the C2 subtype.

The characteristics of LUAD tumor cells are determined by the stromal and immune cells recruited to and activated in the tumor microenvironment (TME). Additionally, the TME in which tumor cells proliferate, develop, and metastasize, is also infiltrated by immune-related molecules and immune cells (36–38). Thus, we examined the differences in the TME between different pentose phosphate-related lncRNAs-based subtypes. A higher degree of immune cell infiltration for most immune cells was found in the C1 subtype relative to the C2 subtype, suggesting that the C1 subtype had a relatively high degree of immune cell infiltration, consistent with the findings of our previous study. The higher immune cell abundance and immune scores for C1 may be a reason for favorable prognoses among these patients. Moreover, some hub immune checkpoints, such as CTLA4, CD274, and CD47, were found to be significantly upregulated in the C1 subtype as compared to the C2 subtype as evidenced by the differences in immune checkpoint expressions. Furthermore, the putative clinical effects of immunotherapy on both molecular subtypes were assessed. The findings suggested that the C1 subtype had a higher score and a greater likelihood for immune escape, which indicated that the patients belonging to the C2 subtype had poorer prognoses, along with a downregulated expression of the immune checkpoints. This further suggested that these patients may be better suited to and benefit more from immunotherapy as compared to those with the C1 subtype. TFs play key regulatory roles in several aspects of tumor progression in multiple cancers, including LUAD. For example, the expression of SOX12 is significantly high in LUAD tissues and is closely associated with the survival and prognoses of patients. It is suggested that SOX12 expression may be an auxiliary predictor for LUAD progression (39). Thus, it is crucial to investigate the activity of TFs in LUAD. Through our analysis, we identified one TF, BACH1, that was consistently and significantly upregulated in the C1 subtype. Moreover, enrichment analysis indicated that BACH1 was involved in the activation of certain pathways and was tightly related to tumor progression and poor prognoses.

In this study, the pentose phosphate correlation-based typing could provide a new perspective for further studies on LUAD, particularly at the level of lncRNAs. A previous study (40) reports the two existing typing criteria for LUAD, namely TCGA subtype and Immune subtype. Comparisons with the existing molecular subtypes revealed no significant differences in the distributions among the subtypes. However, no significant differences in survival curves for these distributions were also observed. Thus, the subtypes proposed in this study were meaningful. We have identified the possible regulatory involvement of pentose phosphate-related lncRNAs.

Nevertheless, further experimental evidence is needed to confirm the findings of this study. For instance, experimental validation of the differences in the expressions of pentose phosphate-related lncRNAs between the C1 and C2 subtypes is needed. Further investigations on the hub immune checkpoints identified herein and if they are significantly different at the transcript and protein levels between the two different subtypes are also warranted. The impact of BACH1 on tumor progression and prognoses needs to be confirmed experimentally. Moreover, the possible mechanism of regulatory interplay also needs to be investigated in the future.

In conclusion, we typed LUAD into two subtypes based on pentose phosphate-related lncRNAs, as C1 and C2 subtypes. The C1 type showed a lower pentose phosphate score and was related to good prognoses, whereas C2 had a higher pentose phosphate score and was associated with poor prognoses. The potential clinical effects differed significantly between the two molecular subtypes, with higher scores associated with clinical effects and a higher likelihood of immune escape in the C1 subtype. In addition, the C1 subtype showed a relatively high degree of immune cell infiltration. There was a significant correlation between subtypes and mutations, as also with genes, including SPTA1, TNR, KEAP1, STK11, and FAT4, of which, the proportion of mutations were significantly higher in the C2 subtype as compared to the C1 type. Finally, the C2 subtype was found to be significantly associated with certain energy metabolic pathways.

## Data Availability Statement

The datasets presented in this study can be found in online repositories. The names of the repository/repositories and accession number(s) can be found in the article/Supplementary Material.

## Author Contributions

CL and YW designed the study and conducted literature review. CL collected the data, analyzed the data, and wrote the first draft of the manuscript. YW interpreted the data and edited the manuscript. Both authors read and approved the manuscript.

## Conflict of Interest

The authors declare that the research was conducted in the absence of any commercial or financial relationships that could be construed as a potential conflict of interest.

## Publisher's Note

All claims expressed in this article are solely those of the authors and do not necessarily represent those of their affiliated organizations, or those of the publisher, the editors and the reviewers. Any product that may be evaluated in this article, or claim that may be made by its manufacturer, is not guaranteed or endorsed by the publisher.

## Supplementary Material

Supplementary Table 1 114 metabolism-related pathways with gene lists.

Supplementary Figure 1 The workflow of this study.

Supplementary Figure 2 The distribution of different clinical features in C1 and C2 subtypes.

Supplementary Figure 3 Differential analysis between C1 and C2 subtypes in TCGA dataset. **(A)** A heatmap of screened DEGs. **(B)** GO analysis of DEGs. **(C)** KEGG analysis of DEGs.

Supplementary Figure 4 Genomic alterations in molecular subtypes in TCGA dataset. **(A)** Differences in Homologous Recombination Defects, Aneuploidy Score, Fraction Altered, Number of Segments, and Tumor Mutation Burden between the two molecular subtypes in TCGA dataset and correlation between them. n-C1 = 186, n-C2 = 30. **(B)** Somatic mutation analysis (Fisher test) of the top 16 mutated genes in both molecular subtypes.
