# Supplementary figures and images for: Identification of Two Subtypes and Prognostic Characteristics of Lung Adenocarcinoma Based on Pentose Phosphate Metabolic Pathway-Related Long Non-coding RNAs
Source: Front Public Health. 2022 Jun 21;10:902445. doi: 10.3389/fpubh.2022.902445 (PMC9253426; doi:10.3389/fpubh.2022.902445)

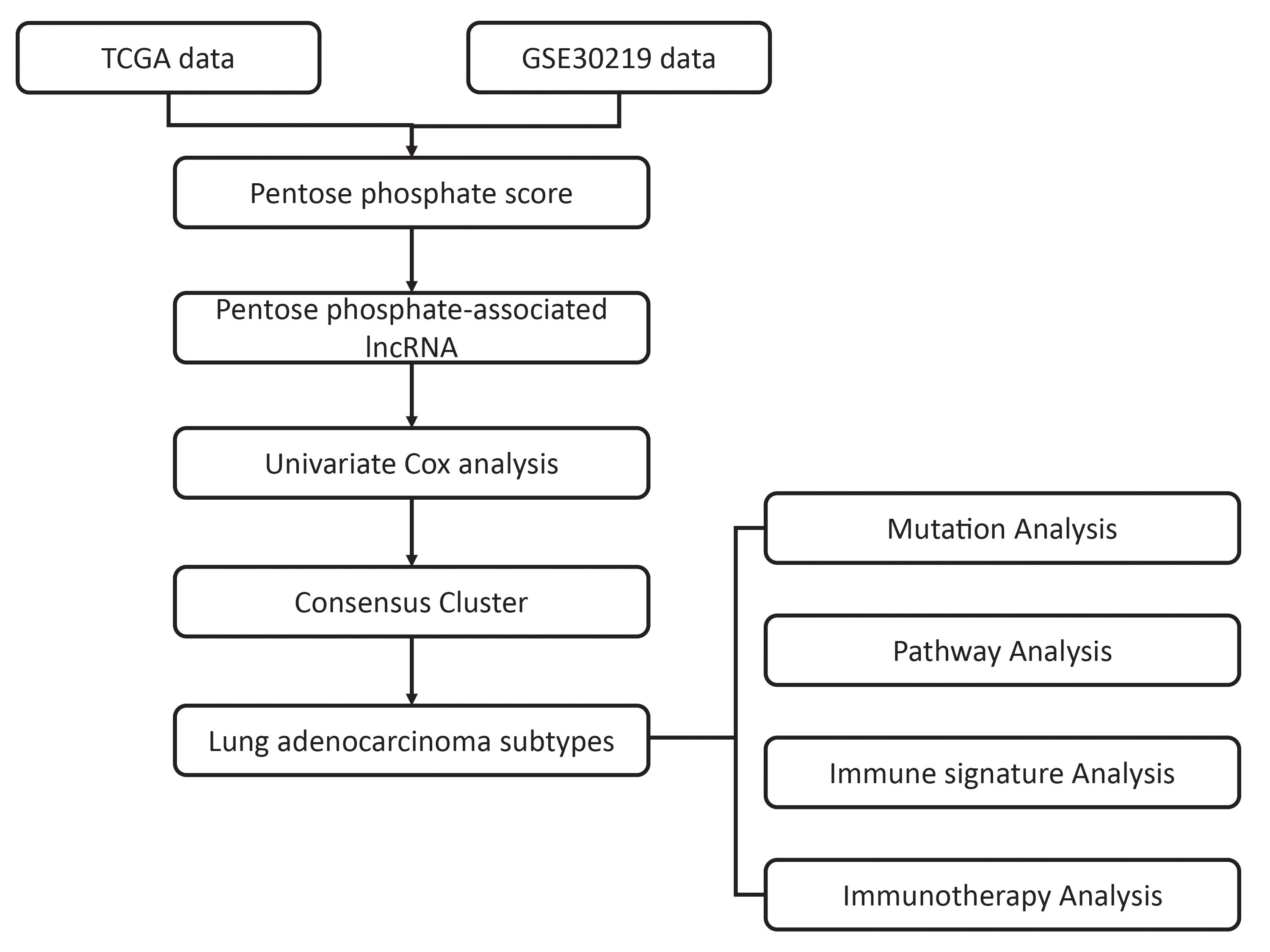

Supplement: Supplementary Figure 1 — The workflow of this study. [file Image_1.JPEG]

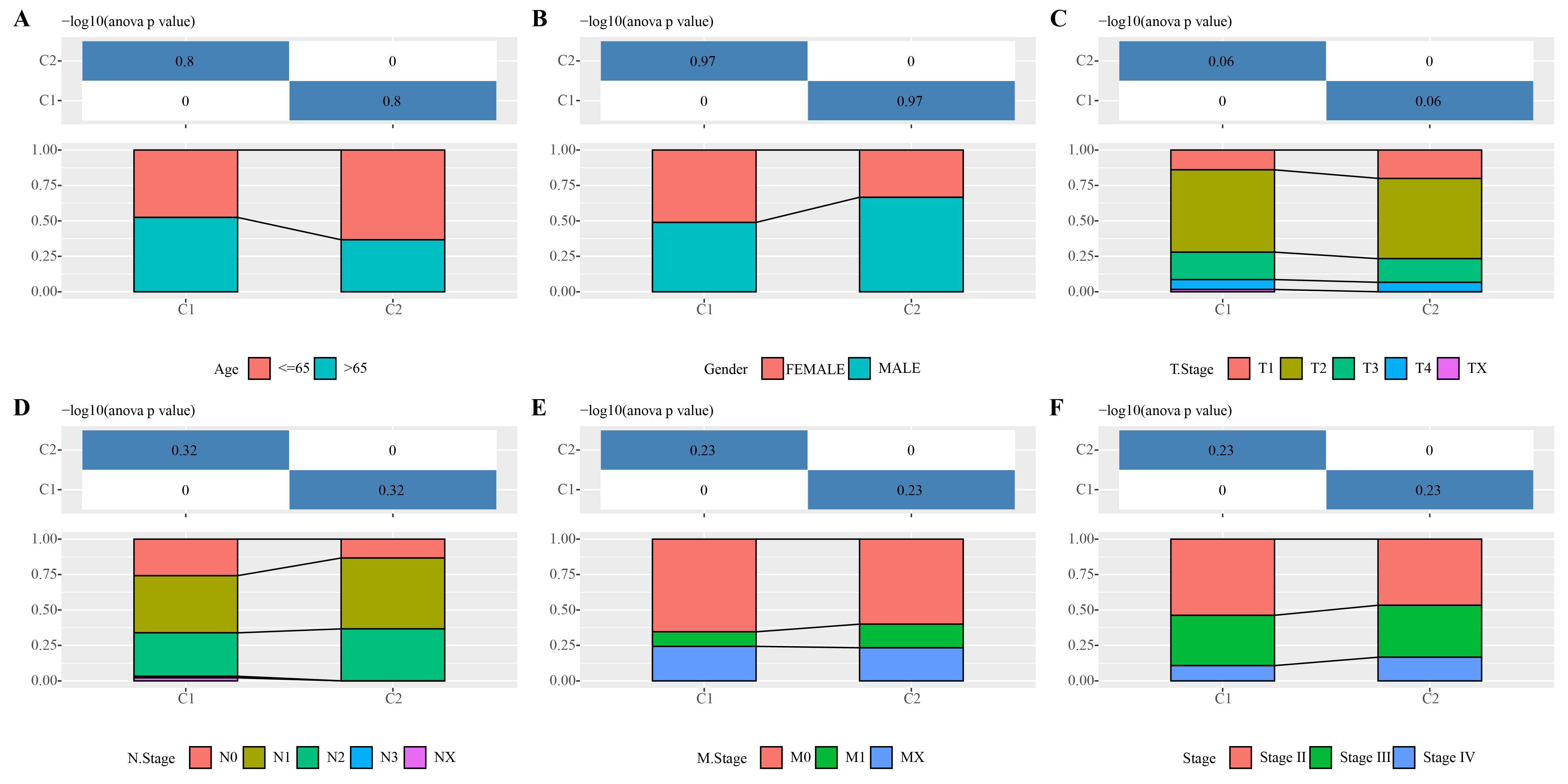

Supplement: Supplementary Figure 2 — The distribution of different clinical features in C1 and C2 subtypes. [file Image_2.JPEG]

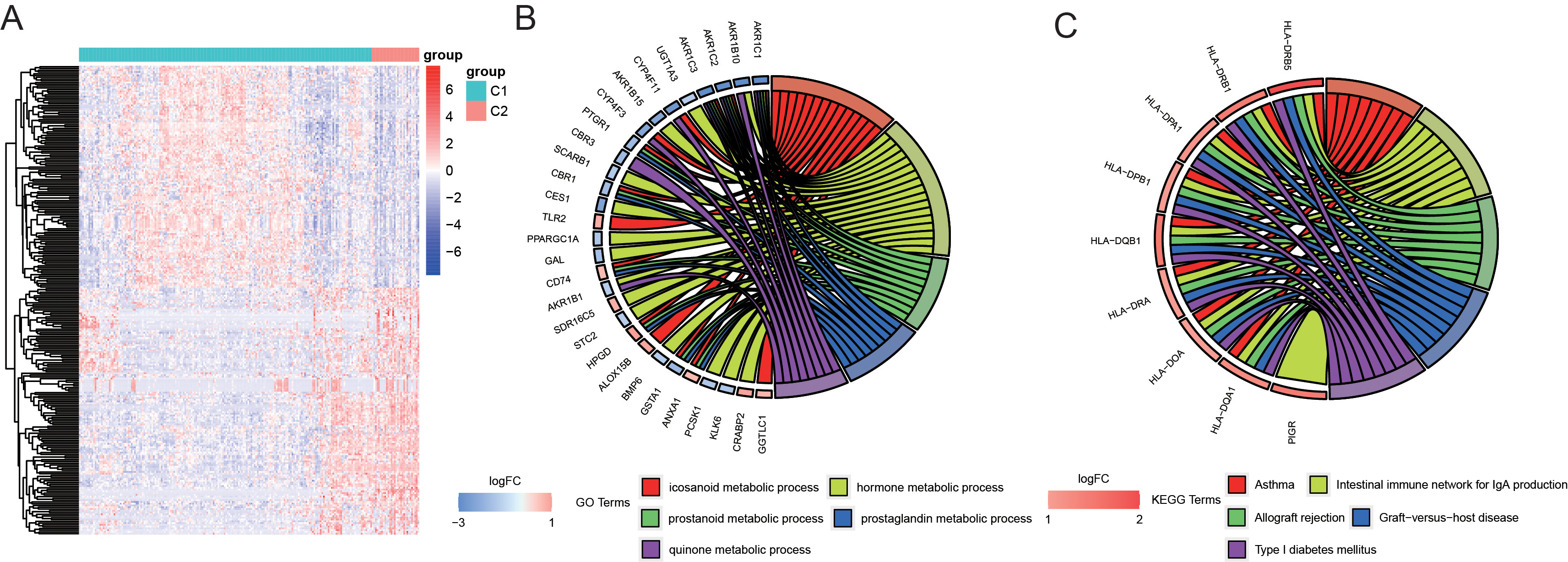

Supplement: Supplementary Figure 3 — Differential analysis between C1 and C2 subtypes in TCGA dataset. (A) A heatmap of screened DEGs. (B) GO analysis of DEGs. (C) KEGG analysis of DEGs. [file Image_3.JPEG]

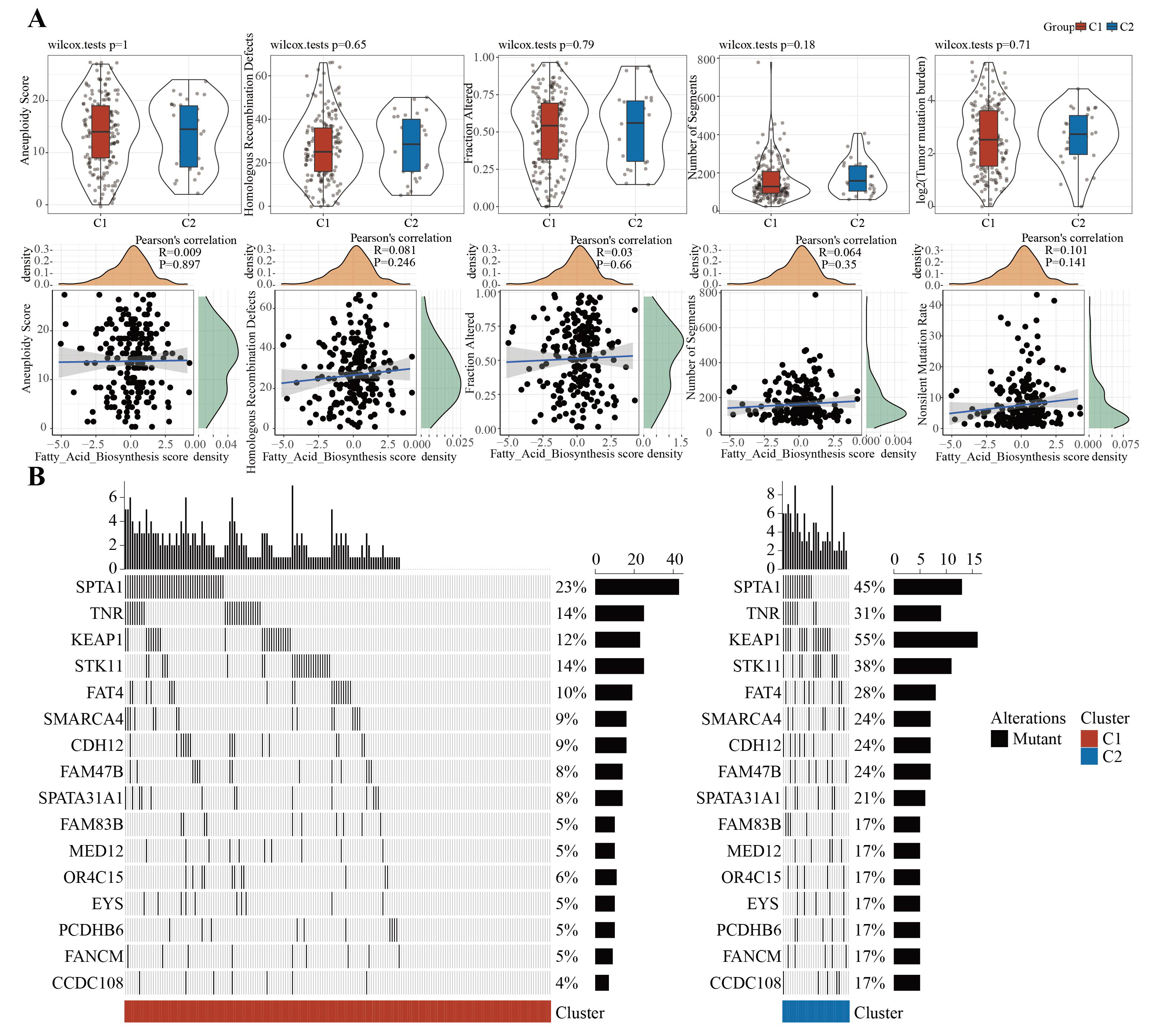

Supplement: Supplementary Figure 4 — Genomic alterations in molecular subtypes in TCGA dataset. (A) Differences in Homologous Recombination Defects, Aneuploidy Score, Fraction Altered, Number of Segments, and Tumor Mutation Burden between the two molecular subtypes in TCGA dataset and correlation between them. n-C1 = 186, n-C2 = 30. (B) Somatic mutation analysis (Fisher test) of the top 16 mutated genes in both molecular subtypes. [file Image_4.JPEG]
